# Visual symptoms associated with refractive errors among Thangka artists of Kathmandu valley

**DOI:** 10.1186/s12886-017-0659-0

**Published:** 2017-12-21

**Authors:** Deepa Dhungel, Gauri Shankar Shrestha

**Affiliations:** 1 Private Practice Optometrist, Kathmandu, Nepal; 2 BP Koirala Lions Center for Ophthalmic Studies, Maharajgunj Medical Campus, Institute of Medicine, Tribhuvan University, Maharajgunj, Kathmandu Nepal

**Keywords:** Astigmatism, Blurred vision, Myopia, Visual symptoms, Watery eye

## Abstract

**Background:**

Prolong near work, especially among people with uncorrected refractive error is considered a potential source of visual symptoms. The present study aims to determine the visual symptoms and the association of those with refractive errors among Thangka artists.

**Methods:**

In a descriptive cross-sectional study, 242 (46.1%) participants of 525 thangka artists examined, with age ranged between 16 years to 39 years which comprised of 112 participants with significant refractive errors and 130 absolutely emmetropic participants, were enrolled from six Thangka painting schools. The visual symptoms were assessed using a structured questionnaire consisting of nine items and scoring from 0 to 6 consecutive scales. The eye examination included detailed anterior and posterior segment examination, objective and subjective refraction, and assessment of heterophoria, vergence and accommodation. Symptoms were presented in percentage and median. Variation in distribution of participants and symptoms was analysed using the Kruskal Wallis test for mean, and the correlation with the Pearson correlation coefficient. A significance level of 0.05 was applied for 95% confidence interval. The majority of participants (65.1%) among refractive error group (REG) were above the age of 30 years, with a male predominance (61.6%), compared to the participants in the normal cohort group (NCG), where majority of them (72.3%) were below 30 years of age (72.3%) and female (51.5%).

**Result:**

Overall, the visual symptoms are high among Thangka artists. However, blurred vision (*p* = 0.003) and dry eye (*p* = 0.004) are higher among the REG than the NCG. Females have slightly higher symptoms than males. Most of the symptoms, such as sore/aching eye (*p* = 0.003), feeling dry (*p* = 0.005) and blurred vision (*p* = 0.02) are significantly associated with astigmatism.

**Conclusion:**

Thangka artists present with significant proportion of refractive error and visual symptoms, especially among females. The most commonly reported symptoms are blurred vision, dry eye and watering of the eye. The visual symptoms are more correlated with astigmatism.

## Background

Visual symptoms have pervasively been reported back from a long time, and have been observed to be associated with various conditions [1–11]. Among them, uncorrected refractive error is commonly considered to be a culprit in reduced visual efficiency [12–14]. Retinal image blur during the near point task, makes performing the near task more difficult and manifests symptoms [15, 16]. Even a low magnitude of hypermetropia manifests symptoms of intermittent blur, headache, fatigue, loss of concentration and inattention [17]. The presence of low to moderate uncorrected astigmatism can also produce a significant increase in asthenopia [18]. With high astigmatism, the ciliary muscles may attempts to correct the error resulting in asthenopia [18, 19]. In the literature, the association between symptoms and uncorrected refractive error is ambiguous. Symptoms associated with refractive error are reported among school children in one study [20], where as it is reported to be unrelated with neither refractive error, nor binocular and accommodative measurements in the other study [21]. The latter finding has been supported by the Neugebauer et al. [22] and Ip et al. [23] studies. No matter what the type and magnitude of refractive error is, the proper refractive correction can alleviate the symptoms of visual strain among the refractive error carriers [15–19].

A thangka is a Tibetan Buddhist painting on cotton, or silk applique, usually depicting a Buddhist deity, scene, or mandala. Thangka artists are exposed to prolonged and repetitive near work using art materials and their job involves precision designs, fine designs and details which require high visual attention and mental concentration. Visual symptoms are common among participants with visually demanding tasks and the presence of uncorrected refractive errors and prolonged near tasks could trigger the visual symptoms among Thangka artists. The present study was conducted to determine the visual symptoms among Thangka artists, and investigate the association of those with refractive errors.

## Methods

### Participants and study design

In a descriptive cross-sectional study, the research setting and population of the study were Thangka painting schools and Thangka artists. There is no exact data available regarding Thangka artists in Kathmandu valley. However, it was assumed that there were around 100 Thangka painting schools operated, and that comprised of around 7000 Thangka artists.

A convenient sampling technique was adopted to select clusters having 50 artists or more. Selection criteria included that the participants were involved in the Thangka painting profession for two years or more, were below the age of 40 years, had no any systemic disease, were not taking any medications for any chronic diseases and provided consent to participate in the study. Any artist who had undergone eye surgeries, had amblyopia and strabismus were excluded from the study. A total of 525 participants participated into the study from six clusters between November 2015 and December 2016. Among the 525 participants, 121 participants (23.04%) had significant refractive error (Myopia ≥ −0.50D, hypermetropia ≥ +0.50D and astigmatism ≥ −0.50D), and of them, 51 participants had worn spectacles. Among the spectacle wearers, only nine participants attained visual acuity better than 0.1 Log MAR and were excluded from analysis. Finally 112 participants qualified for further analysis and were named as the refractive error group (REG). Another 130 participants who were absolutely emmetropic (Myopia <−0.25D, Hypermetropia <+0.25D, Asitgmatism <0.25D), had visual acuity equal to or better than 0.1 log MAR, and had no other accommodative disorders, fusional vergence deficits, heterophoria or heterotropia and tear deficiency conditions, were assigned as a normal cohort group (NCG).

### Informed consent

Meetings with the supervisors were held to describe the purpose of the study, and consent was taken from them to conduct the study. A date for the assessment of symptoms related to their artwork and a detailed eye examination was fixed. After the purpose of the study was explained to each participant, verbal informed consent was obtained from them. The research protocol adhered to the provision of the Declaration of Helsinki for research involving human participants.

### Assessment

#### Structured questionnaire

Visual symptoms were recorded from all the participants, with a structured questionnaire which was developed by Howarth and Costello (1997) [24]. This questionnaire consisted of nine items and each item scoring consisted of 0 to 6 consecutive scales- i.e. no symptom, very slight symptom, slight symptom, mild symptom, moderate symptom, severe symptom and very severe symptom respectively. The higher the scores were, the more were the symptoms.

The English version of the questionnaire was translated to Nepali script and again back translated to English to maintain the consistency of meaning of each sentence. Pretesting was carried out among 10 patients presenting at the OPD of B.P. Koirala Lions Center for Ophthalmic Studies to check the patients’ understanding of the questions.

Each questionnaire was read slowly and clearly for every subject by a researcher, and they were asked for their response that best suited to them. The internal consistency reliability coefficient (Cronbach’s alpha) of 0.74 was revealed on testing collectively among participants with uncorrected refractive error and emmetropic participants.

#### Examination

Uncorrected, presenting and best corrected visual acuity was assessed in each eye with a Log MAR chart at a three meter distance and the finding was recorded. A diagnosis of amblyopia was made if the vision was 6/12 equivalent or worse in one or both eyes after a careful eye examination including funduscopy through a dilated pupil and cycloplegic refraction. Cycloplegic refrction was performed in only those participants who had unstable refractive error, no improvement in visual acuity, and hypermetropia.

Static retinoscopy was performed at 50 cm distance in a moderately illuminated room with the aid of a retinoscope (Heine beta 200) and lens racks, and the findings were recorded after deducting the working distance of +2.00D. A subjective refraction was carried out to determine the spectacle correction which provided optimal visual acuity. For prevalence estimation, significant refractive error was defined as −0.50D or more for myopia, +0.50D or more for hypermetropia and −0.75D or more for astigmatism.

Anterior segment and posterior segment eye examination was carried out with the help of a portable slit lamp and direct ophthalmoscopy (Heine, Beta 200). Cover tests aided by a bar at six meter and 40 cm distances, stereopsis at near with the Titmus fly test, positive and negative fusional vergences with prism bar at six meter and 40 cm distances, the near point of convergence and the amplitude of accommodation with the Royal Air Force ruler, binocular accommodative facility with ±2.00D lens flipper at 40 cm distance, color vision with the Ishihara 38 plates, lag of accommodation at 40 cm distance by the monocular estimation method, the Schirmer test using Whatman-41 filter paper, and blink rate while reading a newspaper without prior information were also carried out.

Participants who had orthophoria, exophoria (equal to or less than 4pd), esophoria (equal to or less than 2pd), positive fusional vergence (break at 21pd for near and 19pd for distance), amplitude of accommodation (greater than or equal to 2.00D than the age match as calculated by Hofsetters formula), lag of accommodation (+0.75 D to +1.25D), near point of convergence (equal to or less than 10 cm), accommodative facility (equal to or more than 10 cycles per minute), color vision test (less than six plates error in counting), stereopsis (60 s of arc or better), wetting of the Schirmer strip (equal to or greater than 10 mm in 5 min) and a good blink rate (equal to or greater than12 blink per minutes) were considered normal.

#### Data analysis

All the data were reviewed and analysed using the Statistical Package for Social Sciences version 16.0. Descriptive statistics were presented as frequency, percentage, means and standard deviation. Ocular-visual symptoms were presented in median. Variation in distribution of participants and symptoms was analysed using the Kruskal Wallis test. Similarly age and gender prevalence of refractive error was analysed using Chi-square test. Correlation between mean of total symptom score and myopia and astigmatism was presented by the Pearson correlation coefficient. In all analyses, a significance level of 0.05 was applied at a 95% confidence interval.

## Results

Age and sex distribution of participants in the REG and the NCG are presented in Table 1. The majority of participants in the REG represents the age group above 30 years (Median: 33 years), whereas the majority of participants in the NCG represents the age group below 30 years (Median: 26 years), the variation being significant (*p* = 0.000). Mean age is 30.98 years (SD ± 6.89) in the REG and 26.17 (SD ± 6.21) in the NCG. Males have predominated (61.6%) the distribution in the REG compared to the NCG, where almost equitable gender distribution can be noted.

**Table 1:** Age and sex distribution of subjects in the Refractive Error Group (REG) and the Normal Cohort Group (NCG)

Characteristics	Group variables	Refractive Error Group No (%)	Normal Cohort Group No (%)	
Age group (Years)	16–20	7 (6.3)	29 (22.3)	0.000*
	21-25	12 (10.7)	39 (30.0)	
	26–30	20 (17.9)	26 (20.0)	
	31–35	39 (34.8)	22 (16.9)	
	36–40	34 (30.3)	14 (10.8)	
Mean age ± SD		30.98 ± 6.89	26.17 ± 6.21	0.000**
Sex	Males	69 (61.6)	63 (48.5)	0.12^#^
	Females	43 (38.4)	67 (51.5)	
Total		112 (100)	130 (100)	

*Significant for *p*-value <0.05 by Kruskal Wallis test

**Significant for *p*-value <0.05 by independent sample t-test

^#^Significant for *p*-value <0.05 by Chi-square test

Distribution of visual symptoms is presented in Table 2. Visual symptoms reported are relatively high among Thangka artists. While comparing the findings between the REG and the NCG, dry eye (*p* = 0.004) among external symptoms and blurred vision (*p* = 0.003) among internal symptoms are reported higher among the REG. In contrast, the severity of symptom of watering is significantly higher among the NCG (*p* = 0.02).

**Table 2:** Distribution of the visual symptoms in the Refractive Error Group (REG) and the Normal Cohort Group (NCG)

Symptoms	Symptoms reported for at least once in percentage		Percentage symptom scores		Median		*p*-value*
Items	REG	NCG	REG	NCG	REG	NCG	
Internal symptoms							
Tired eye	52.5	50.5	12.3	11.9	2	2	0.20
Sore or aching eye	61.0	55.8	12.6	12.9	2	3	0.35
Blurred vision	69.5	47.4	15.2	12.5	3	1	0.003
Double vision	23.7	20	8.2	8.1	1	1	0.95
External symptoms							
Dry eye	44.1	36.8	11.8	9.6	1	1	0.004
Burning eye	55.9	52.6	12.9	13.5	2	2	0.86
rritated eye	47.5	45.3	10.5	10.6	2	1	0.25
Watering eye	37.3	49.5	9.0	12.6	1	1	0.02
General visual discomfort	18.6	25.3	7.5	8.3	1	1	0.80

*REG* Refractive error group, *NCG* normal cohort group

*Significant at *p* = value < 0.05 by Kruskal Wallis Test

Age and sex distribution of percentage symptom scores among the REG and the NCG is presented in Table 3. There was a greater variation in reported symptoms by gender than the age group, and they are mostly internal symptoms, such as tired eye (*p* = 0.01), sore eye (*p* = 0.02) in females in the NCG and double vision (p = 0.02) in females in the REG. However, blurred vision is noted significantly high among participants with the age group 21–25 years (*p* < 0.05) and males (*p* < 0.01). Regarding external symptoms, burning eye is recorded significantly higher among participants of the age group 31–35 years (*p* < 0.05) and females (*p* < 0.05). In the REG, there has been a significant age variation for watering eye (*p* = 0.007), and males have reported significant higher symptom of watering eye in the NCG (*p* < 0.05) than the REG.

**Table 3:** Age and sex distribution of symptom scores in the Refractive Error Group (REG) and the Normal Cohort Group (NCG)

Symptoms	Subject groups	Percentage symptom score								
		Age group in years						Sex		
		16–20	21–25	26–30	31–35	36–40	*P* ^#^	Males	Females	*P* ^#^
Internal symptoms										
Tired eye	REG	13.0	12.3	16.2	9.7	11.2	0.11	11.5	12.1	0.27
	NCG	13.6	14.0	11.7	9.3	14.4	0.43	**11.1**	**13.5**	**0.01**
Sore or aching eye	REG	17.4	12.9	14.0	11.8	10.1	0.06	10.7	14.2	0.25
	NCG	15.0	12.4	12.3	13.8	12.3	0.82	**12.2**	**13.9**	**0.02**
Blurred vision	REG	21.8	14.8*	16.2	14.5	18.2	0.87	17.9**	14.0	0.66
	NCG	11.6	10.7	12.0	13.8	12.3	0.52	12.5	11.7	0.62
Double vision	REG	8.7	7.7	6.0	9.5	8.5	0.55	**7.9**	**8.9**	**0.02**
	NCG	9.0	7.8	6.0	9.1	8.4	0.56	8.2	7.9	0.71
External symptoms										
Dry eye	REG	5.8	6.5	12.0	11.8	10.8	0.20	11.9	8.9	0.18
	NCG	10.3	9.1	12.9	11.0	9.8	0.31	9.9	11.2	0.07
Burning eye	REG	14.5	14.2	12.0	15.4*	12.5	0.09	13.1	14.9*	0.80
	NCG	11.6	12.4	15.7	11.9	11.3	0.12	13.4	12.1	0.55
Irritated eye	REG	5.8	9.0	12.0	9.7	11.5	0.35	9.8	10.8	0.14
	NCG	7.3	11.6	11.4	11.5	11.3	0.07	10.6	10.9	0.28
Watering eye	REG	**7.2**	**12.9**	6.0	**10.6**	**9.8**	**0.007**	9.2	10.2	0.11
	NCG	10.6	14.2	10.0*	12.1	10.8	0.20	12.6*	11.2	0.76
General visual discomfort	REG	5.8	9.7	5.6	7.0	7.4	0.14	8.0	6.0	0.80
	NCG	11.0	7.8	8.0	7.5	9.4	0.62	9.4	7.7	0.67

*REG* Refractive error group, *NCG* normal cohort group

^#^significant at *p*-value <0.05 by Kruskal Wallis test

*Significant at *p*-value <0.05 by Kruskal Wallis test

**Significant at *p*-value < 0.01 by Kruskal Wallis test

Of the 112 participants with refractive errors, myopia (between −0.50 and −6.00D) has become the most common refractive error among 83 participants (74.1%), followed by astigmatism (between −0.50 and −2.00D) in 22 participants (19.6%) and hypermetropia (between +0.50 and +2.00D) in seven participants (6.3%). Myopia between −0.50D and −3.00D is reported among 78 participants, whereas myopia between −3.25 and −6.00D is noted only among five participants with mean refractive error of −1.50 (SD 1.3). Similarly, mean refractive errors for astigmatism is observed at −1.1 (SD 0.3), and for hypermetropia at +0.8 (SD 0.2).

**Fig. 1:**
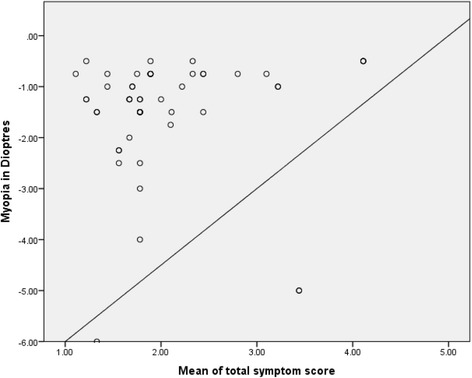
Correlation between myopia and mean of total symptom score

**Fig. 2:**
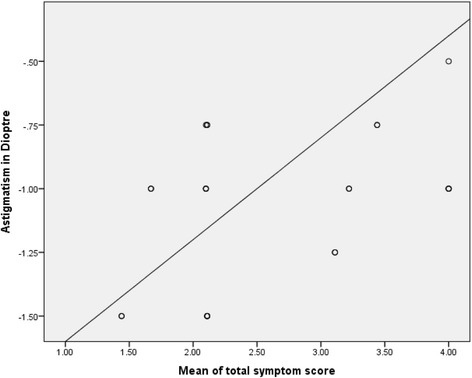
Correlation between astigmatism and mean of total symptom score

The association between visual symptoms and refractive error in the REG is presented in Table 4. By comparing symptoms among participants with myopia and astigmatism, sore/aching eye (*p* = 0.003) and blurred vision (*p* = 0.02) among internal symptoms and feeling dry (*p* = 0.005) among external symptoms have been significantly associated with astigmatism, whereas general visual discomfort (*p* = 0.04) is observed higher among myopic participants. The correlation (Fig. 1) between myopia and mean of total symptom score shows a feeble relationship (*r* = 0.038, *p* = 0.787) where as correlation between astigmatism and mean of total symptom score (Fig. 2) has been high (*r* = 0.445, *p* = 0.029).

**Table 4:** Association between visual symptoms and refractive error in the Refractive Error Group

Symptoms	Percentage symptom score			
	Types of refractive error			
	Myopia	Hypermetropia	Astigmatism	^#^ *p*-value*
Internal symptoms				
Tired eye	12.5	8.0	12.8	0.43
Sore or aching eye	12.5	5.3	14.9	0.003
Blurred vision	14.6	16.0	17.0	0.02
Double vision	8.9	5.3	6.6	0.72
External Symptoms				
Dry eye	11.1	8.0	15.3	0.005
Burning eye	12.5	18.7	12.5	0.41
Irritated eye	10.3	10.7	11.1	0.078
Watering eye	9.1	20	5.6	0.93
General visual discomfort	8.5	8.0	4.2	0.04

^#^comparison between myopia and astigmatism

*Significant at *p*-value <0.05 by Kruskal Wallis test

## Discussion

The present study reports the visual symptoms and their association with refractive errors, particularly myopia and astigmatism among Thangka artists.

Regarding spectacle wear, 70 participants (57.9%) among the REG didn’t wear spectacle and another 42 participants (34.7%) wore an improper correction. This finding of not wearing spectacle is significantly higher than the previous study from Nepal among video display terminal (VDT) users, where refractive error was uncorrected in only 14 participants (18.4%) who presented with asthenopia in clinical settings [25]. In our study the majority of participants have a small magnitude of refractive errors and are mostly myopic (Myopia ≤ −2.00D in 60 participants; Myopia > −2.00 ≤ −6.00D in 11 participants; astigmatism ≤ −1.50 in 34 participants; hypermetropia ≤ +1.00D in 7 participants). The VDT users were the participants who presented to clinical setting owing to the visual symptoms. There is a possibility that they were more aware regarding need of periodic eye examination or symptomatic than Thangka artists.

The overall visual symptoms reported for at least once (Table 2) among the REG (45.6%) are slightly higher than the NCG (42.6%), The mean difference between the total symptom scores for the REG (2.16 ± 0.74) and the NCG (1.98 ± 1.08) is noted at only 0.18, which is statistically insignificant (*t* = 1.17, *p* = 0.24). However, among the items, percentage symptom scores for blurring of vision (*p* = 0.001) and dry eye (*p* = 0.004) are significantly greater for the REG than the NCG. All the participants in the REG had either uncorrected or improperly corrected refractive error. It is clear from the findings why there was a significant reporting of blurred vision in the REG. In contrast, the symptom of watery eye was significantly higher among the NCG (*p* = 0.02) than the REG. Many studies [18, 25–28] reported symptoms associated with near work, but the type of symptom was different depending upon the nature of work. Untimanon et al. (2008) have reported eye burning (26.6%), eye pain (14.0%) and eye irritation (11.2%) among electronic and jeweler workers [26]. Similarly, Amelia et al., (2010) have reported a high prevalence of asthenopia at 69.7% among computer using students as a result of accommodative insufficiency [27]. Salve has reported not only the high prevalence of symptoms (65.2%), but also a high level of job related complaints among jeweler manufacturing workers [28]. In the Wajuihian study, headache was the most prevalent symptom that accounted for 40.8% of all symptoms among school age children [18]. Shrestha et al. (2011) have reported tired eye as the most common symptoms reported by 88.2% with headache being the most intense symptom represented by 13.3% of total symptom score among computer users attending to the hospital [25]. The findings of these studies are not absolutely related to refractive error alone except in the Wajuihian study. We have not included systemic symptoms like headache, should pain, neck pain and back pain, which was reported in the other studies. The difference in asthenopia across the studies can be influenced by classification, types and recording of asthenopia. Moreover, they are recorded in different age group, occupation and research settings. It is clear from the findings that visual symptoms are significantly higher among all Thangka artists irrespective of refractive errors, though the symptoms tend towards the REG indicating prolonged near and minute work manifests visual symptoms.

For the age and sex distribution of symptom scores, there are little difference in symptoms between the REG and the NCG (Table 3), especially among females of the NCG for tired eye (*p* = 0.01) and sore eye (*p* = 0.02), and females of the REG for double vision (*p* = 0.02). Similarly, the symptom of burning eye was also higher (*p* < 0.05) among females of the REG than the NCG. However, blurred vision has been reported higher (*p* < 0.01) among males of the REG than the NCG. Literature suggests most symptoms were more frequent in females than in males suggesting that females may have more symptoms and more frequently than do male patients, or that more females than males were more likely to report symptoms more frequently [20]. Our study also suggests that the symptoms are slightly inclined towards females than males. Related to these findings, looking downward gaze for near work and decreased blinking has greater likelihood of manifesting dry eye symptoms which includes burning and tearing. Also females in menstrual cycle or on contraceptives would also report more dry eye symptoms. Though, in our study, those participants with reduced blink rate and the Schirmer value have been excluded, blink rate was not assessed while they were painting. Also, the Schirmer value doesn’t represent the wide spectrum of dry eye analysis. Likewise, we did not assess the history related to the contraceptive use and periodic symptoms of dryness among females, especially regarding the period of menstrual cycle. The Wajuihian study has also reported three symptoms viz headache, photophobia and redness more associated with females [18].

Literature have reported low hyperopia was the reason for poor reading and low academic performance and associated with asthenopic symptoms like intermittent blur, headache, fatigue, loss of concentration, eye strain and even inattention in some children [29, 30]. However, hypermetropia being present in only seven participants, we didn’t entertain them for further analysis. Considering the fact that the hypermetropia is more relevant when considering the accommodating demand at near task for Thangka artists, further study may be warranted regarding assessing association between the symptoms and hyperopic refractive error. As we compared symptoms between myopia and astigmatism, sore/aching eye (*p* = 0.003), dry eye (*p* = 0.005) and blurred vision (*p* = 0.02) have significantly associated with astigmatism. It is clear that uncorrected astigmatism may cause continuous adjustment in the ciliary muscles in trying to compensate for the error, therefore leads to asthenopia. However, general visual discomfort (*p* = 0.04) has associated with myopia. This finding aligns with the findings of previous studies for association between symptoms and astigmatism [18]. However, headache was the most associated symptom with astigmatism [18.30]. Even a low magnitude of astigmatism is the most common refractive cause of ocular headaches in young individuals [31]. In our study, we have included participants having astigmatism equal to or greater than −0.50D. Our study has a limitation in the part that systemic symptoms were not included in the study to compare with refractive error. For this reason, our findings may not be directly comparable to the previous reports.

Against-the-rule and oblique astigmatism are suggestive to produce more blur and symptoms than with-the-rule [3, 31]. In our study, the majority of the astigmatic participants (72.7%) have with-the-rule astigmatism followed by against-the rule astigmatism (18.2%) and minimal cases of oblique astigmatism (9.1%).

The present study is conducted in just a small segment of occupational groups and sample size is even smaller for the comparison of components of refractive errors with the symptoms. The systemic symptoms are not included in the questionnaire. Generalization of the findings may be limited because of all these factors.

## Conclusion

Finally, it is clear that a significant proportion of Thangka artists have refractive error and visual symptoms especially among females. The mostly commonly reported symptoms are blurred vision and dry eye among participants with refractive error and watering eye among normal participants. The visual symptoms appear to be more correlated with astigmatism than myopia.

## References

[1] American National Standards Institute. American National Standard Practice for office lighting, ANSI/IESNA RP-1-1993. New York: American National Standards Institute, 1993.

[2] Jaschinski-Kruza W, Schweflinghaus W (1992). Relations between dark accommodation and psychosomatic symptoms. Ophthalmic Physiol Opt.

[3] Wiggins NP, Daum KM (1991). Visual discomfort and astigmatic refractive errors in VDT use. J Am Optom Assoc.

[4] Wiggins NP, Daum KM, Snyder CA (1992). Effects of residual astigmatism in contact lens wear on visual discomfort in VDT use. J Am Optom Assoc.

[5] Bachman WG (1992). Computer-specific spectacle lens design preference of presbyopic operators. J Occup Med.

[6] Butzon SP, Sheedy JE, Nilsen E (2002). The efficacy of computer glasses in reduction of computer worker symptoms. Optometry.

[7] Sheedy JE (1992). Reading performance and visual comfort on a high resolution monitor compared to a VGA monitor. J Electronic Imaging.

[8] Sheedy JE, McCarthy M (1994). Reading performance and visual comfort with scale to gray compared with black and white scanned print. Displays.

[9] Sheedy JE, Kang JM, Ota WT, Sheedy JE, Shaw-McMinn P (2002). Vertical eye gaze position: effect on task performance and visual comfort. Diagnosing and treating computer-related vision problems.

[10] Wilkins AJ, Nimmo-Smith I, Slater AI, Bedocs L (1989). Fluorescent lighting, headaches and eyestrain. Lighting Res Tech.

[11] Toda I, Fujishima H, Tsubota K (1993). Ocular fatigue is the major symptom of dry eye. Acta Ophthalmol.

[12] Scheiman M, Wick B (2008). Clinical management of binocular vision: Heterophoric, accommodative and eye movement disorders.

[13] Rutstein RP, Daum KM, Amos JF (1988). Accommodative spasm: a study of 17 cases. J Am Optom Assoc.

[14] Evans BJW (2008). Optometric prescribing for decompensated heterophoria. Optom Pract.

[15] Rosenfield M (2011). Computer vision syndrome: a review of ocular causes and potential treatments. Ophthalmic Physiol Opt.

[16] Shrestha GS, Dhungel D (2017). Vision related problems in visually demanding occupations: a mini review. JOJ Ophthal.

[17] Abdi S, Rydberg A (2005). Asthenopia in school children, orthoptic and ophthalmological findings and treatment. Doc Ophthal.

[18] Wajuihian SO (2015). Frequency of asthenopia and its association with refractive error. Afr Vision Eye Health.

[19] Abdi S, Lennerstrand G, Pansell T, Rydberg A (2008). Orthoptic findings and asthenopia in a population of Swedish schoolchildren aged 6 to 16 years. Strabismus.

[20] Hendricks TJW, De Brabandar J, Horst FVD, Hendrikse F (2007). Relationship between habitual refractive errors and headache complaints in school children. Optom Vis Sci.

[21] Collins MJ, Brown B, Bowman KJ, Carkeet A (1990). Vision screening and symptoms among VDT users. Clin Exp Optom.

[22] Neugebauer A, Fricke J, Russmann W (1992). Asthenopia: frequency and objective findings. Ger J Ophthalmol.

[23] Ip JM, Robaei D, Rochtchina E, Mitchell P (2006). Prevalence of eye disorders in young children with eyestrain complaints. Am J Ophthal.

[24] Howarth PA, Costello PJ. The development of visual test battery for virtual reality users. Contemporary. Ergonomics. 1997:276–81.

[25] Shrestha GS, Mohamed FN, Shah DN (2011). Visual problems among video display terminal users in Nepal. J Optom.

[26] Untimanon O, Pacharatrakul W, Boonmeepong K (2006). Visual problems among electronic and jewelry workers in Thailand. J Occup Health.

[27] Amalia H, Suardana GG, Artini W (2010). Accommodative insufficiency as cause of asthenopia in computer using students. Universa. Medicina.

[28] Salve UR (2015). Vision related problems among the workers engaged in jeweler manufacturing. Indian J Occup Environ Med.

[29] Ip JM, Robaei D, Kifley A, Wang JJ, Rose KA, Mitchel P (2008). Prevalence of hyperopia and association with eye findings in 6- and 12-year-olds. Ophthalmology.

[30] Marasini S, Khadka J, Sthapit PR, Sharma R, Prasad BJ (2012). Ocular morbidity on headache ruled out of systemic causes: a prevalence study carried out at a community based hospital in Nepal. J Optomal.

[31] O’Leary C, Evans BJW (2003). Criteria for prescribing optometric interventions: literature review and practitioner survey. Ophthal Physiol Opt.

